# Republican Theory and the EU: Emergency Laws and Constitutional Challenges

**DOI:** 10.1007/s42439-021-00044-3

**Published:** 2021-10-05

**Authors:** E. Herlin-Karnell

**Affiliations:** University of Gothenburg, Gothenburg, Sweden

**Keywords:** EU, Emergency laws, Republican theory, Coercion, Judicial review

## Abstract

The COVID-19 pandemic has raised many intriguing questions both in the EU and globally, from the critical task of safeguarding lives to technical legal issues about competences to regulate health as well as the boundaries of emergency laws. This paper is interested in the connection between non-domination theory and the EU’s constitutional structure in the context of emergency laws. A key theme of the paper is that risk and emergencies are nothing new in an EU context, but concepts used by the legislator in a wide range of policy areas which give rise to a number of constitutional challenges. The paper sketches out the main characteristics of non-domination and republication theory and addresses the question of how and why the notion of non-domination may be useful for understanding the EU constitutionalism venture in the framework of risk and emergency laws.

## Introduction

The COVID-19 pandemic has raised many intriguing questions both in the EU and globally, from the critical task of safeguarding lives to technical legal issues about competences to regulate health as well as the boundaries of emergency laws.^1^ In this paper, I examine the connection between non-domination theory and the EU’s constitutional structure in the context of emergency laws. Coercion is a central concept in legal and political philosophy, often used to define the role of the state, and the EU is interesting in this regard as an example of a supranational and mixed intergovernmental entity. Do emergency powers give rise to coercive powers and how are they justified? A system where coercion may be identified needs to be justified according to classic constitutional canons. This gives rise to the question of what the exact meaning of restricting rights is and when and to what extent emergency laws and derogations represent justified coercion at all. To some extent, this is of course the well-trodden albeit fascinating legal debate on the use of derogations and the meaning of proportionality for balancing rights.^2^ But these questions could also be understood from the prism of non-domination and constitutionalism, and it is the latter aspect that is the focus of the current paper.

While republican theory has had a revival in both legal studies and political theory lately,^3^ it remains a largely unexplored theory in the framework of law and especially in the EU framework.^4^ Republican theory has many interrelated strands.^5^ For example, Philip Pettit has advocated a “neo-republicanism” with a focus on freedom as non-domination.^6^ For Pettit, freedom as non-domination incorporates the liberal constitutionalist commitment to basic rights, the rule of law and democratic governance, and checks and balances.^7^ This makes it appealing in a legal context as it fits the structure of law of a justice-based system tracking down on arbitrary domination. But it is, as just noted, not the only interpretation of republican theory, which in fact entails many different strands. In this paper, the concept of freedom as non-domination and the focus on arbitrary domination are understood as an important branch of republication theory and as being of relevance for constitutional theory. The notion of freedom in the legal meaning and in the sense of open borders and mobility has always been a very central feature in EU law and has constituted one of the hallmarks of EU integration. Freedom in this sense has both curtailed and enhanced the Union and its Member States.^8^ Freedom in EU positive law – the internal market context for example – entails (inter alia) the freedom to engage in cross-border activities without extra national burdens and has constituted one of the main rationales of the creation of EU citizenship.^9^ Likewise, the idea of freedom is essential in the “Area of Freedom, Security and Justice” policy, but here, freedom is part of the security and justice venture of the EU and is connected to the architectural structure and as such reflects mainly negative freedom. Perhaps it should be mentioned here that some scholars have questioned the focus on constitutional law as part of the republican project.^10^ Specifically, they have argued that a theorist’s defense of, for example, constitutional safeguards by itself tells us nothing about the particular conception of freedom that the theorist subscribes to. While this paper may be guilty of this, it is not its aim to define freedom; instead, it is interested in the impact of republican theory in the context of EU law.

The paper has two parts: a doctrinal EU legal part and a more theory-oriented part that discusses non-domination and constitutional theory in the light of the real-time problems of emergency laws. The first part will briefly map the EU legal landscape of emergency legislation in EU law and thereby aims to paint the background picture vis-à-vis the legal questions that are represented in this area. As will be shown, risk and emergencies are nothing new in an EU context and are concepts used by the legislator in a wide range of policy areas. The classic underlying question in this regard is to what extent the constitutional system of the EU is equipped to deal with emergency situations and the possible tensions with regard to the proper application of the rule of law and proportionality. Moreover, an associated matter that is discussed in this paper is what the boundaries of emergency legislation are and how we should understand these questions from a constitutional perspective. The second part of the paper discusses emergencies in the context of coercion and republican theory as well as how a constitutionalist vision and lens could help us understand EU constitutional law and the issue of emergency laws and judicial review. The paper sketches out the main characteristics of non-domination and republication theory and addresses the question of how and why non-domination theory may be useful for studying the EU constitutionalism venture. In the final section, I summarize my argument.

I make three claims in the paper. Firstly, and as noted above, risk regulation and emergencies are nothing new in EU law, but it may be helpful to briefly spell out how the legal framework works and that this is needed in order to test the legal implications of risk and emergencies in practice. Secondly, non-domination theory is useful in legal contexts as many of the main constitutional questions facing the EU such as the reach of the proportionality principle and the right to judicial review concern the question of how to secure a free and equal system that is non-dominating yet inclusive and integrates EU law. Thirdly, republican theory or non-domination is not exactly the same as the rule of law debate. The paper sets out to explain why this is the case and why it matters in the context of emergency laws and EU constitutional theory. Let me start with the legal question of what exactly risk and emergency mean in EU law and how it is perceived, before exploring the potential relevance of non-domination theory in EU constitutionalism.

## EU Law, Risk, and Emergency

While COVID-19 represents an unprecedented challenge, the phenomenon of risk regulation is far from new in EU law. Emergencies and risk regulation have been a hot topic in EU law for a long time, from various food-related crises to security emergencies and the fight against terrorism.^11^ The concept of risk regulation forms part of primary EU law. Take for example the protection of the environment (Articles 191–192 *Treaty of the Functioning of the European Union*), consumer protection (169 *Treaty of the Functioning of the European Union*), (Article 114 *Treaty of the Functioning of the European Union*), and market regulation which are central policy areas in the internal market.^12^ In this area, the EU shares competences with the Member States, but where EU action often “pre-empts” Member States from acting.^13^ It is also an area where the EU engages in both “risk management” and “risk assessment”, where policy alternatives are weighed in consultation with interested parties and appropriate prevention and control options are selected.^14^ Of special interest in EU law has been the precautionary principle that has been around since the 2000s.^15^ The precautionary principle has been frequently invoked in food crises, chemicals regulation, and environmental protection cases more generally.^16^ The precautionary principle is currently also discussed in the framework of the new green European deal for sustainability.^17^ It is a tool to manage risks, uncertainties, and where the importance of predicting the future is central.

In this context, for example, the European Agency for Environmental Matters has recently argued that the precautionary principle is not used to its full potential.^18^ In short, the precautionary principle can be used by either the EU legislator (as a cause to take action if the “risk” prompts action to be taken before the danger has occurred) or by Member States.^19^ The precautionary principle means that decisions can be made before there is concrete evidence of a risk that needs to be countered; it is future oriented and developed in the context of environmental law and the need to fight emerging environmental catastrophes and food-related risks.^20^ The interrelationship between risk regulation and the precautionary principle is rather complex, and the latter is being applied in many different, though interrelated, contexts.^21^ Moreover, the precautionary principle is mostly on the agenda in connection with environmental protection and is especially important in relation the regulation of circumstances of scientific uncertainty.^22^ In addition, for example, the notion of risk is important in the fight against money laundering, terrorism financing, and security regulation, where the notion of risk is essential.^23^ Hence, the EU has a competence to legislate on anti-money laundering legislation and terrorist financing (Article 114 TFEU and Article 83 TFEU). Consequently, one of the EU objectives is to make sure that the internal market is not distorted by various Member State responses to combat risks and emergency in various policy areas.^24^ More specifically, the EU will seek to actively support efforts to address new and emerging risks at global level, and this concern matters ranging from environmental law to financial crimes to the fight against terrorism.^25^

In the context of the responses to fight COVID-19, the EU Commission has, among other things, recently issued a communication and an action plan concerning how to manage the crisis.^26^ Important issues raised in connection with the COVID-19 crisis concern, inter alia, the reach of public health measures and the management of EU borders. Does the EU have the legal authority to regulate public health? For one thing, public health is one of the possible derogations that Member States can invoke in order to set aside EU law, meaning that Member States (Article 52 *Treaty of the Functioning of the European Union*) can derogate from their EU law obligations if the derogations in question are deemed proportionate and non-discriminatory. Moreover, in EU law, public health is mainly a Member State competence and constitutes one of the possible derogations from EU free movement law, Article 52 TFEU. In short, EU Member States can derogate from free movement on the basis of public policy, public security, or public health. Responses to the COVID-19 crisis have already had effects in other legal areas, i.e., beyond the sphere of health care, where risk regulation is central. For example, the EU Commission’s recent communication on the prevention of money laundering and terrorist financing is interesting here as the Commission states that there is an increase in criminal activities in the context of the COVID-19 pandemic. The communication is a good example of the importance of risk monitoring in EU law and its global dimension.

The large scale of the COVID crisis puts solidarity to the test. The corona bonds and financial turmoil discussion, as well as closed borders, are all unprecedented challenges, even if the phenomenon of emergencies and risks are not new in EU law as previously mentioned. The EU has established a recovery instrument of €750 billion, at the time of writing highly controversial, which will boost the EU budget with new financing raised on the financial markets for 2021–2024. The corona bonds discussion challenges solidarity and trust in the EU, which is interesting in itself as solidarity has turned out to be such a challenging and contested concept yet crucial for the operation of EU law.

The EU recently celebrated 30 years of having its own emergency number – 112.^27^ At the time of writing, there is a battle over COVID-19 vaccine, and there is a crisis of solidarity (again) in the EU.^28^ The COVID-19 crisis has led to a temporary, partial shutdown of European and global political life. So the EU and its Member States have had to adopt emergency measures to preserve the health of the citizens and prevent a collapse of the economy. With regard to emergencies, the EU itself does not have an emergency provision, and the EU allows also for various derogations for Member States to allow for national exceptions from EU law on a legal basis that is proportionate and non-arbitrary. However, risk and emergencies are nothing new in the EU. The interesting question raised in this paper, as I will try to argue, concerns the use of derogations. In line with EU law, there is a possibility of public security derogations within the internal market framework (e.g. Article 36 TFEU), while national security is a competence for the Member States (Article 4 TEU). The European Convention on Human Rights (ECHR) emergency provision, Article 15, allows state signatories to derogate from their human rights obligations under states of emergency. Article 15 (1) ECHR stipulates that in time of war or other public emergency threatening the life of the nation, any high contracting party may take measures derogating from its obligations under this Convention to the extent strictly required by the exigencies of the situation, provided that such measures are not inconsistent with its other obligations under international law.

Emergency powers are known by a number of different synonyms, where exception to the rule is one component of emergency.^29^ Today, it is common to speak of a permanent state of emergency with regard to, for example, the fight against terrorism post 9/11 in the USA. In terms of state of exception as part of the legal set up, Israel is an example of a state that has had a state of exception since 1948.^30^ Oren Gross explains how, in the case of Israel, the temporary regime became a permanent feature in the life of the state, and it remains an integral part of the Israeli legal system. Similarly, until August 2005, Northern Ireland was subject to emergency rule for a combined period of over 30 years.^31^ While the US used a war-based model to fight terrorism in the wake of 9/11, emergency government became the norm.^32^ The picture has looked slightly different in Europe as the EU has adopted a criminal law and administrative sanctions approach. The EU mainly uses a risk-based approach increasingly linked to both the financial regulation area and that of environmental protection and with a focus on exactly the precautionary principle. Moreover, while security is predominantly a national competence (Article 4 TEU, which states that national security remains the responsibility of each Member State), the EU has, in fact, adopted a number of security measures. Those security measures severely blur the distinction between the EU and national security, and even global security.^33^

The main question regarding derogations in EU law is often that of proportionality and non-discrimination. Yet the vocabulary of derogations in the EU internal market law is not the only area where the language of derogations is frequently used. The EU constitutional structure and the question as to what extent it should be possible to apply derogations at all are interesting.

### What Derogations?

Derogations in EU law are probably as old as EU law itself. Member States have according to the Treaty an option to opt out of EU law under certain circumstances. The idea that Member States can derogate from constitutional duties, if it can be justified, is a common feature of human rights law. For example, Member States can invoke exceptions for, e.g., national security, national identity, and public health. All derogations from EU law, however, have to pass the proportionality test (and non-discrimination) to count as a valid justification.^34^ The ECHR framework has a similar make-up, albeit focused on human rights protection of course. Yet, unlike the EU law rights framework, the ECHR does not grant direct effects of rights and is mainly directed at focusing on whether the state is granted a certain margin of appreciation.^35^ The question of to what extent Member States have a margin of appreciation is of course an ongoing issue, both legal and political. Likewise, in the context of the EU, the principle of proportionality appears to play a key role regarding both the scope and the limit of rights. For example, Article 52 (1) of the Charter sets out some important exceptions to the application of the Charter as a whole.^36^ Limitations of rights, that is a derogation, can be done when they are necessary and genuinely meet objectives of general interest recognized by the Union or the need to protect the rights and freedoms of others. Admittedly, such a limitation is not unique to the EU: Article 5 ECHR has a similar limitation of the presumption of freedom based upon when it is necessary and justified. So, when is it justified then and is there any clear connection between emergencies and derogations?

As for the ECHR system, a core set of the Convention’s provisions are, as is well-known, not possible to derogate from and are absolute, such as the right to life and the right not to be subjected to torture. EU law on the other hand is not as specific on whether the rights are absolute or not but appears to allow more derogations but stipulates that ECHR will always constitute the minimum standard (Article 52.3).

Perhaps the term *derogation* itself indicates an abnormal nature of emergencies where the regime is a product of the dialectic of dichotomy between normalcy and emergency.^37^ Moreover, Gross has argued that it is imperative to ensure that “emergency” and “crisis” do not become expedient governmental tools used to facilitate the violation of individual rights. To that end, derogation from such rights is made possible only in the most extreme circumstances. Derogation measures last only temporarily, and their ultimate purpose ought to be that of bringing about a rapid return to “normalcy”.^38^ Derogations are usually contingent on proportionality analyses. The idea of proportionality is one of the most central principles of constitutional law. For example, for Aharon Barak, proportionality links up with the question of justice because “There is always law (domestic or international) according to which the state must act. There are no black-holes”.^39^ The principle of proportionality allows for some stability, until the real work of legislation and deliberation begins. Hence, the merits of proportionality outweigh its downsides, as it could help improve the democratic shortcomings where the legislator has failed to do so, assuming that we agree that this is what a democratic system should do.^40^ Any interference or derogation must pursue a legitimate aim connected to a common public good, and this concern is captured, at least in part, in the existing methodologies of constitutional and human rights courts.^41^

The idea of rights-based constitutional adjudication seems consistent with a political concept of rights, accommodating the fact of disagreement about rights.^42^ The discrepancy between the use of emergencies as a legislative tactic and that of the rule of law is very interesting and fairly well-documented. For example, Ronald Dworkin linked the question of derogations to right as trumps. But if rights can be derogated from, are they still trumps? In this context, Dworkin called for a clearer connection between the threats that are said to justify serious limits on rights on the one hand and the harm we may present in the course of dealing with the suspect, prompting some scholars to argue that the case of terrorism has come to complicate the simplicity of the trumps as rights theory.^43^

In order to clarify the type of derogations we are discussing, this paper is interested in the link between possible domination, emergency laws, and the meaning of coercion in the context of judicial review.

## Emergency Laws, Coercion, and Judicial Power

Carl Schmitt infamously argued that the rule of law cannot constrain state action during state of exception and consequently that sovereign is he or she who decides on the exception.^44^ As noted above, the EU itself does not have an emergency provision; instead, it has the public security derogations within the internal market framework. It could certainly be challenged whether the emergency powers that Schmitt discussed in the context of a nation state could be extended to the EU supranational level at all.^45^ For example, one could argue that the fact that the EU allows derogations but does not have emergency provisions in the same way as nation states use it indicates that the emergency provision cannot be used to the EU itself. The EU is not a state, but it is nonetheless to a large extent a supranational organization with some state-like powers such as increased competences in criminal law matters and tax as a result of the Lisbon Treaty. What does it tell us about the relation between sovereignty and emergency powers? Here, we encounter an interesting issue in EU law, namely, to what extent EU law is still authoritative without a system of effective enforcement in place, or at least largely dependent on national law for enforcing EU law (which is often a problem due to various national reasons).

Furthermore, are emergency laws or the application of the risk principle an expression of coercion or black holes and is there then still law to derogate from? The boundaries of coercion are connected to the question of the role and function of the state. But what does it mean to refer to coercion? Jacob Weinrib has argued that in a legal system, private rights are secured through public coercion.^46^ While coercion may involve the imposition of sanctions, force seems slightly different in that actors subjected to force are compelled through physical power. Frederick Shauer recently stated that simply identifying coercion as the characteristic and arguably distinguishing feature of law are a fairly limited endeavor.^47^For him, law is vital because it requires people to set aside their own preferences. Indeed, Dworkin used coercion as an example of how the very point of legal regulation is to provide a moral justification for the coercive enforcement of legal norms and that some sort of backup in terms of coercion is conceptually necessary.^48^

What then when the derogation from a right amount to coercion and when is it justified?

In constitutional and fiduciary theory, states may derogate from their human rights commitments in emergencies only where such measures are strictly necessary to satisfy their overarching fiduciary obligation to guarantee security and equal freedom.^49^ Interestingly, Evan Criddle and Evan Decent-Fox have suggested that one way to answer the question regarding the use of emergency provisions may be to turn to a view from fiduciary law:

‘On the fiduciary theory, states bear an obligation to safeguard their subjects’ equal freedom during emergencies—even if this requires derogation from some human rights norms such as the freedoms of expression, movement, and peaceable assembly. Non-peremptory human rights norms are subject to derogation in contexts where the strict observance of these norms would conflict with the state’s overarching fiduciary obligation to guarantee subjects’ secure and equal freedom. But states must also refrain from taking measures in emergencies that would simply replace private domination with public domination.^50^

For Criddle and Fox-Decent, the fiduciary theory adopts an intermediate position between the view that all human rights are absolute and the so-called Schmittian-inspired view that states may abrogate human rights unilaterally in emergencies.^51^ But most importantly, they argue that a fiduciary theory monitors an international law’s emergency constitution in the sense that it allows human rights to provide relevant standards even when these rights are subject to derogation. In this sense, the margin of appreciation in human rights law becomes a double proportionality test, both regarding the justification for the derogation in question as well as the proportionality of the derogation in the individual case.

The point here is that the fiduciary model confirms a non-domination-oriented view that fits squarely with the constitutionalist viewpoint that of a right to justification and judicial review.^52^ This is interesting as often the very purpose of emergency provisions is that they cannot be challenged in real time. The question for our purpose is to what extent a non-domination theory lens could offer anything new in this regard?

## Republican Theory and the EU: What it Is and Why it Is Important

As noted at the outset of the paper, in the last decade or two, there has been a revival in republican theory. This section will briefly scan some of the main debate on republicanism and the EU, what it is and why it is important. While there is a dispute within the republican camp, regarding, for example, the relationship between liberalism and non-domination, for Pettit, for instance, a non-arbitrary law does not compromise freedom.^53^ As Christine Laborde explains it, the non-domination theory incorporates the liberal constitutional commitment to basic rights, the rule of law, democratic governance, and checks and balances in its definition.^54^ It should perhaps be stressed here that I do not understand non-domination as being exactly the same as the rule of law, even though the importance of securing that arbitrary domination does not exist has a clear procedural element to it.^55^ There is clearly a connection between the rule of law and non-domination. A crucial debate in legal philosophy about the rule of law concerns exactly the relationship between the ideal of the rule of law and other values and purposes.^56^ The non-arbitrariness test is central here.

The republican idea of non-domination is generally considered the foremost yardstick for testing the level of freedom in a society,^57^ where the authority of power deprived of reason is an unbound form of domination.^58^ This debate can be taken further in different directions. In short, a Kantian reading of non-domination understood as non-interference is strictly meaning a public law right to freedom.^59^ Others argue for an understanding of freedom that involves positive duties for the legislator to also legislate on welfare issues and that is also relevant in the public law context. To some extent, the debate is also reflected in Isaiah Berlin classic idea of “two conceptions of liberty”, namely, a positive and a negative notion of freedom.^60^ With the negative conception of freedom, people are free simply to the extent that their choices are not interfered with. This is in line with the liberalism expressed by John Stuart Mill, for example, and has been the dominant view of freedom.^61^ The positive conception is trickier: it is freedom in the sense that groups or individuals exercise self-control or self-mastery.

The classic definition of neo-republicanism is an interest or a good that everyone desires and because freedom is seen as non-domination.^62^ In order to identity the notion of domination, Pettit uses the well-known slave-master relationship as the prime example of “unfreedom”. The criteria for non-domination and its relation to freedom and equality are, however, still debatable. Despite its contested nature, the conception of domination, according to some scholars, is commonly marked by three important features^63^: (1) domination is seen as a problem in specific social relationships; (2) domination is understood as being structure based, meaning that it is not a particular outcome of domination that matters; and (3) domination is identified as the use of arbitrary interference or alien control. In Pettit’s vocabulary, domination means something along the lines of “living under any agent who possesses the capacity to interfere with choices in an arbitrary manner”.^64^ In short, the non-domination view means that you are objectively under the powers of others if arbitrary will is dictating your life and you stand to them as the slave stands to a benevolent master.^65^ In the context of the debate between liberalism, neo-republicanism, and Kantian conceptions of freedom and non-interference, respectively, further others have argued that freedom as independence is superior to its liberal and republican rivals.^66^

In the context of the EU, Richard Bellamy has recently distinguished between a free person where as he argues that the more traditional notion of freedom is relevant, i.e., negative freedom, and that of a free state which as he argues concerns circumstances of legitimacy.^67^ The standard of legitimacy must therefore incorporate mechanisms for accountability that are both more robust and more inclusive than that provided by the consent of democratic states. Moreover, for him, the EU should be seen as a republican association that is designed to overcome the possibility of the mutual domination between states while providing mechanisms for their securing certain global goods, not least through the reciprocal recognition of citizenship.^68^ For Bellamy, republican theory represents a modified version of statism, which emphasizes the importance of the nation state while also stressing that sovereign states must recognize cosmopolitan obligations towards each other and establish institutional structures that ensure non-domination for all.^69^ Still, Bellamy’s idea seems to be that of semi-statism, although he calls it a statist cosmopolitanism. This can potentially be contrasted with the viewpoint consisting of using the EU as a means to agree, implement, and enforce rules that apply in common, which is a way to improve the legitimacy of political decision-making at the EU level and beyond that which can be achieved by unilateral action.^70^ The legitimacy question has been highlighted by others who argue that the EU subscribes to a flawed concept of popular sovereignty, prioritizing justice over legitimacy.^71^ On this view, the source of legitimation is critical responsiveness, confirming and confronting a critical theory of legitimacy beyond the sphere of a moralized conception of freedom and justice.

Both non-domination theory and legitimacy are relevant also in the critical theory of Rainer Forst.^72^ In brief, from Forst’s viewpoint, securing freedom must be reciprocally and generally justifiable, but no one has the authority to fabricate these justifications for you because you are an autonomous agent of justification.^73^ Securing the autonomy of the individual is one of the values protected by the rule of law. As Joseph Raz reminds us, people can predict how power will be exercised over them and therefore can plan and pursue long-term goals.^74^ Non-domination theory seems to target this in multiple ways both at the individual, state, and meta level.

For a Kantian, a system of equal freedom is one in which each person is free individually or co-operatively, in order to set their own purposes and be the authors of the law.^75^ In a Rousseauean-Kantian fashion, freedom is the autonomy to be the co-author of the norms that bind you and non-interference.^76^ Yet for Forst, under non-domination theory as advocated by Pettit, citizens are “law-checkers” interested in securing their freedom of choice, but they are not law makers.^77^ Moreover, as Arthur Ripstein explains, the notion of freedom in the republican sense means that a state can be fully realized only if the freedom of obeying no other law than that to which the citizen has given its consent. This means that you are only independent and not dominated if you are the object and subject of the law at the same time. For a Kantian, independence is not a good to be promoted: it is a constraint on the conduct of others, imposed by the fact that each person is entitled to be their own master.^78^

The EU seems perhaps to be a mixed case in this regard: citizens are not directly law makers, and they can use EU law as a trump card against a Member State; the doctrines of (inter alia) supremacy and direct effect give precedent to EU law over conflicting national law and in the absence of a justification.^79^ Avoiding arbitrariness through a right to justification and non-domination test so that all are treated as free and equal is a central question when discussing the boundaries of justice. Hence, arbitrary rule is the rule of some people over others without legitimate reason.^80^ Forst describes this as an order of justification, where, as already noted, non-domination is set to avoid arbitrariness. This order of justification is especially interesting in an EU constitutional theory setting as non-domination may have two immediate effects in this context. On the one hand, non-domination may mean that the EU should do more to protect its citizens especially in cases where the Member States are not doing enough and hence citizens ought not to be dominated by their own Member State. On the other hand, the EU’s institutions may be the source of domination. In those cases, national constitutional law in a pluralism-oriented EU protects from domination.

For sure, non-domination theory as advocated by Pettit emphasizes the importance of multilateral international arrangements and endorses the ideal of globalized sovereignty.^81^ Also John Rawls famously stipulated principles of international justice designed to safeguard the international freedom and equality of peoples.^82^ Both have been criticized for being surprisingly statist oriented as they do not develop the international dimension of non-domination or its relationship to the duty of assistance.^83^ This Kantian republican theme of justice through law is developed in the work by Ripstein and, as mentioned above, is interesting in the context of the EU.^84^ For example, Kant rejected a world state in favor of a “pacific league” or “permanent congress” of states and that states provide for a condition of public right.^85^ According to Ripstein (citing Kant), nobody has standing to coerce others into joining a world state and that a larger federation is itself not a mandatory end.^86^ The EU then has characteristics of a cosmopolitan legal order, but is neither totally Kantian nor un-Kantian. Dignity and autonomy are crucial concepts in EU law, but the EU has developed as something more than a cosmopolitan union focusing on peace as the EU has a complex supranational system in many various policies across EU law.^87^

The question of who the author of the law is, as mentioned above, of course intrinsic in Kantian theory. Likewise, it is crucial for non-domination theory. For example, Pettit has claimed that the non-arbitrariness of public decisions depends in part on the ability of citizens to contest them.^88^ Constitutional law is about bounded government and about checks and balances. Constitutionalism is not only equated with a demand for limited government, i.e., the checks and balances constrain, but also entails more positive obligations to provide for the well-being of its citizens.^89^ This is the reason, for example, the concepts of equality, judicial review, fairness, and justice are constitutional principles.^90^ The idea of non-domination seems to entail a more all-encompassing notion of freedom as opposed to a strictly negative or positive conception of it.^91^ In any case, perhaps there seems to be a dissonance here between that of outcome-oriented matters and non-instrumental questions. The distinction between instrumental and non-instrumental is sometimes difficult to draw in law, as it consists of a mixture of the two, and both are needed to realize rights in practice. While the existence of domination may be easier to identify than what exactly non-domination is, non-domination is set to identify exactly the importance of paying attention to the non-instrumental value of constitutional rights.^92^

If the non-domination criteria are a broad one that is tailored to the constitutional structure of a system, involving due process rights and the rule of law, how does this play out in the context of constitutional safeguards and the right of judicial review?

## Non-Domination and Judicial Review

The right of judicial review has been characterized as a non-instrumental notion.^93^ In this regard, the more serious a limitation of rights is, the more intense the review engaged in by the court should be.^94^ In constitutional theory, courts, like legislatures, are representative institutions.^95^ Courts derive their authority from a chain of legitimation that is ultimately anchored in “the people”.^96^ Thus, one of the characteristics of the rule of law is the right to judicial review.

As Mattias Kumm emphasizes: “Besides the requirement of legality – any limitations suffered by the individual must be prescribed by law – the proportionality requirement lies at the heart of determining whether an infringement of the scope of a right is justified”.^97^ If states are to treat all as free and equal, judicial review in terms of the procedure used and the outcomes generated needs to be demonstratively justifiable to those burdened by them as free and equal persons. The question of emergency laws is interesting in the context of the function of judicial review. Consider Kantian theory again, for example. As Ripstein has explained, it is a matter of public right that a state is under a positive obligation to take steps to secure, maintain, and improve a rightful condition.^98^

The question of non-domination and judicial review is also pertinent from an emergency point of view. Let us return to the question of risk and emergency laws as discussed above. With regard to the COVID-19 crisis, as Kim Lane Sheppele explains, among the emergency measures adopted in the case of Hungary during the pandemic in 2020 was a criminalization of people who disrupt the emergency laws, and the emergency laws give a huge discretionary power to detain anyone who challenges what the government is doing in the name of the threat and risk.^99^ Connected to this is the existing rule of law debate in the EU and the associated debate on “backsliding” – or regression – and the current challenges to the rule of law in certain EU Member States such as Poland and Hungary.^100^

With regard to national divergencies in the fight against the pandemic, the EU Commission has, among other things, issued a communication stressing that border management at the EU level is key.^101^ The new tracing apps for monitoring the spread of COVID-19 are a novelty with regard to the crisis management at the EU level. The EU Commission states that it has been assessing the effectiveness, security, privacy, and data protection aspects of digital solutions to address the crisis and that contact tracing apps, if well-coordinated and fully compliant with EU rules, can play a key role in all phases of crisis management.^102^

An issue raised by the COVID-19 crisis is the increasing use of digital data to track citizens. For example, Israel and South Korea, and EU countries such as Germany and Italy, are already using this method (at the time of writing).^103^ The issue has already partially been subject to judicial review. In Israel, the Israeli Supreme Court has held that any monitoring of mobile phone data needs to be backed by adequate provisions in law. It has also stipulated that the tracking system was ultra vires.^104^ The question of outsourcing is highly relevant from a constitutional perspective as it involves private actors taking public law-related decisions. This constitutes a challenge with regard to both EU data protection and the right to dignity and privacy as well as the question of how these issues can be contested in a court. Relevant here are obviously the General Data Protection Regulation^105^ and the eDirective.^106^ In the *Schrems II* case, the EU Court of Justice held that data protection rules in the EU, such as the GDPR, apply to the transfer of personal data for commercial purposes by an economic operator established in a Member State regardless of whether that data is liable to be processed by the authorities of a third country for the purposes of public security, defense, and state security.^107^

The extension of public norms to private actors poses a challenge to representation.^108^ The justification for the state’s power depends on its ability to act impartially in the name of all. Is this debate on the legitimacy of private actors more complex at the EU than at the nation state level? Contemporary political theorists and legal theorists almost exclusively discuss the nation state.^109^ While surveillance mechanisms in place are sometimes unavoidable in order to safeguard a secure society, it raises many questions as to what the limits are. What are the rights of the individual in case of mistakes? How can EU privacy rules and data protection be upheld? What is considered proportionate? These are some of the interesting questions that need to be asked. In recent years, there has been a massive outsourcing to private companies as key players in security regulation both at the EU level and globally. Private companies like Twitter, Facebook, and Google as well as airlines and banks are required to monitor suspicious activities or data generally. Key features of due process may therefore be endangered, as it is often not clear how an individual could be guaranteed the right to judicial review.^110^ When powers are outsourced, both the accountability and the democratic dimension are challenged. This gives rise to the question of how legitimacy can be secured in these matters and connects to the debate on non-domination theory. It is here that the constitutional structure is essential for safeguarding the rule of law and where checks and balances as well as a non-instrumental value of the law as such are important, following both the Kantian and neo-republication path in this regard.

Outsourcing (to private actors) and the use of artificial intelligence (AI) are increasingly common in EU regulation, not least with regard to security and emergency matters. While various surveillance mechanisms in place are sometimes unavoidable in order to secure a secure society, it raises many questions. This is one type of emergency legislation often associated with counter-terrorism legislation. These kinds of measures are used in new areas, most currently the COVID-19 crisis. This gives rise to further questions regarding the boundaries of arbitrary domination, the meaning of coercion, the right to judicial review, and the question of justification. Hence, it is a real test case of theory.

Finally, in order to determine more precisely when the competence of courts is supported by democratic legitimacy, we may employ the three key values of democracy: accountability, participation, and equality.^111^ In this regard, the more serious a limitation of rights is, the more intense the review engaged in by the court should be.^112^ While Article 19 TEU and Article 47 EU Charter set out the right to effective judicial protection and a fair and public hearing (in a similar way as the ECHR regime), how it works in practice across the EU as well as the broader normative implications of the use of derogations remains largely unexplored.

## Concluding Remarks

Non-domination and republican theory have enjoyed renewed focus as a constitutional law theory. On the one hand, while Kantian theory is sometimes criticized for focusing almost exclusively on coercion and not taking into account all the different elements of justice when constructing freedom,^113^ liberal theory, on the other hand, may not tell us all the answers with regard to substance and transnational questions. Against this background, non-domination theory as understood in constitutionalism could offer a third holistic option. The EU has a constitutional structure, where the rule of law, democracy, and human rights are central. Can non-domination achieve anything in this area? Where does emergency and risk fit in here? How can a balance be achieved between necessary derogations and the risk of a normalization of derogations? These are crucial constitutional questions that this paper has aimed at sketching answers to.

Non-domination theory then is an overall framework for how to tackle arbitrary measures through a constitutionalism lens. It is not exactly the same as the rule of law, although the rule of law forms part of what it means to refer to a non-dominating legal system. What is key here is the non-arbitrary test and the need for a justifactory legal system. Borrowing from Yitzhak Benbaji,^114^ it is a semi-Kantian theory. Dignity and the right to due process are key, and emergency laws may sometimes be needed if subject to justifications. But only a republican-based framework and taking into account many interelated questions that fully recognize how complex the EU constitutional structure and its interaction with the regulary sphere has become, could achieve a truly just system (even in times of emergency).

## Footnotes

1 In the EU legal context, see, e.g., A Alemanno 2020, 307–16 and I Goldner Lang 2021, 1–24.

2 E.g., A Barak 2012.

3 P Pettit 2012, I Shapiro 2012, A Harel 2014, E Daly 2015, Daly 2019.

4 See, e.g., A Kocharov 2017. R Bellamy 2019, E Herlin-Karnell 2019.

5 C Laborde 2013.

6 P Pettit 1997.

7 Ibid.

8 J Weiler 1991.

9 See, e.g., A Somek 2008, I Ward 2009.

10 C List & L Valentini, 2016, 1043–1074.

11 Risks and crisis measures have also been frequently discussed, inter alia, in the context of the financial crisis of 2008, the migration crisis of 2015 as well as terror attacks in Europe in recent years 2015/2017.

12 Article 4 *Treaty of the Functioning of the European Union and* Article 4 (3) *Treaty of the European Union*.

13 See, e.g., Weatherill 2016.

14 E.g., EU general risk assessment methodology COM (2013) 76, available at https://ec.europa.eu/docsroom/documents/17107?locale=en. Accessed 31 May 2021.

15 Among many commentators, e.g. L Fisher et al 2006.

16 Ibid.

17 The European Environment State and Outlook 2020, available at https://www.eea.europa.eu/publications/soer-2020. Accessed 31 May 2021.

18 Ibid.

19 E.g., P Craig, 2006 p 721, A Burgess, et al. 2016, F Malgosia et al., 2011.

20 Ibid.

21 Among many commentators, e.g., J Torriti 2007.

22 Any risk assessment at the EU level must also comply with the principle of proportionality. See already the EU Communication on the precautionary principle, COM (2000) available at https://eur-lex.europa.eu/LexUriServ/LexUriServ.do?uri=COM%3A2000%3A0001%3AFIN%3AEN%3APDF. Accessed 31 May 2021.

23 See, e.g., Directive 2018/843 on anti-money laundering and terrorist financing.

24 Commission communication (2020) 2800, action plan for a comprehensive Union policy on preventing money laundering and terrorist financing.

25 See Directive (EU) 2018/843 of the European Parliament and of the Council of 30 May 2018 amending Directive (EU) 2015/849 on the prevention of the use of the financial system for the purposes of money laundering or terrorist financing and amending Directives 2009/138/EC and 2013/36/EU.

26 COM (2020) 1753 final, COVID-19, guidelines for border management measures to protect health and ensure the availability of goods and essential services.

27 https://ec.europa.eu/digital-single-market/en/news/eu-emergency-number-112-marks-30th-anniversary. Accessed 31 May 2021.

28 www.nytimes.com/2021/02/26/world/europe/EU-vaccine-hunt.html?searchResultPosition=8. Accessed 31 May 2021.

29 Greene 2018, D Dyzenhaus 2006.

30 D Scharia 2015, 91.

31 O Gross 2006, 74–88, O Gross 1998, 438–499.

32 Gross 2006 ibid.

33 Directive 2017/541 on combating terrorism, and replacing Council Framework Decision 2002/475/JHA and amending Council Decision 2005/671/JHA. See also Directive of the European Parliament and of the Council on the Prevention of the Use of the Financial System for the Purpose of Money Laundering and Terrorist Financing, Directive (EU) 2015/849, Directive 2013/40/EU, L 218/8 Directive on attacks against information systems.

34 On proportionality in EU law, see, e.g., Zglinski 2020, Tridimas 2012, Craig 2012.

35 Y Shaney 2005, 907–940.

36 Article 52 (1) EU Charter states: Any limitation on the exercise of the rights and freedoms recognised by this Charter must be provided for by law and respect the essence of those rights and freedoms. Subject to the principle of proportionality, limitations may be made only if they are necessary and genuinely meet objectives of general interest recognised by the Union or the need to protect the rights and freedoms of others.

37 Gross (2006).

38 Ibid.

39 A Barak 2005, 287–308.

40 M Kumm 2010, 141–175. The scholarship on the pros and cons of proportionality is vast, see, e.g., A Barak 2012, Klatt and Meister 2012, K Möller 2012, R Alexy 2002, G Webber 2009, M Cohen-Eliya and Porrat 2013, M Klatt 2019.

41 E Daly 2015, 289–316.

42 Ibid.

43 R Dworkin 2006, R Diab 2019, 233–257.

44 C Schmitt 1985, see also, e.g., EJ. Criddle and Fox-Decent 2012, 39–87.

45 Thanks to Enzo Rossi for pointing this out.

46 J Weinrib 2014.

47 F Schauer 2015.

48 R Dworkin 1986, 93, see also discussion in K E Himma 2020 preface and Ch. 1.

49 E Criddle and Fox-Decent 2012.

50 Ibid, 41.

51 Ibid, 41–42.

52 M Kumm 2010.

53 P Pettit 2012.

54 C Laborde 2013.

55 That is part of the rule of law and due process guarantee.

56 E.g., S Taekema 2021 33–66, discussing the debate between Raz and Waldron.

57 P Pettit 2012.

58 C Corradetti 2019, 33–54.

59 A Ripstein 2009, 15.

60 I Berlin 1958.

61 JS Mill, *On Liberty* (1869), which has formed much of modern criminal law based on the harm principle (according to the harm principle, the only purpose for which power can rightfully be exercised over any other member of a civilized community against their will is to prevent harm being done to others). For an overview, see, e.g., AP Simester and A von Hirsch, 201.

62 F Schubbert, (2015), at 440.

63 Schuppert, ibid.

64 Pettit 2012.

65 N Kolodny 2019.

66 List & Valentini 2016.

67 Bellamy 2019 61**–**64.

68 Ibid.

69 See also M Patberg 2020.

70 S Weatherill 2016, 7.

71 J-P Beetz & E Rossi, 2017, 22**–**41.

72 Forst 2017.

73 Ibid 159.

74 J. Raz 1979. See also the discussion in NW Barber 2018, 87**–**105.

75 Ibid 33.

76 R Forst 2020.

77 R Forst 2017, see also E Daly 2017.

78 Ripstein 2009, at 15**–**16.

79 See, e.g., Weatherill 2016.

80 R Forst 2012.

81 L Ypi 2014.

82 A Banai 2015 see also S Caney 2005.

83 L Ypi, ibid (n 72), K Chor-Tan 2016.

84 Ripstein 2009, 323 fn 51.

85 Ibid.

86 Ibid.

87 On cosmopolitan structure and the EU see, e.g., A Stone Sweet & C Ryan, 2018, B Wolthuis and L Corrias, 2021, P Elefteriades 2020.

88 Pettit 1997. See also on constitutionalism and republican theory in Y Dawood 2008.

89 NW Barber 2018, Ch 1.

90 Ibid.

91 See, e.g., I Carter 1999, focusing on negative freedom.

92 See the discussion in Harel 2014.

93 Harel ibid.

94 Ibid.

95 M Kumm 2019, 281–291.

96 Ibid.

97 M Kumm 2010, on courts and emergency, see, e.g., L Henderson 2018.

98 Ripstein 2009, 26–27.

99 K Lane Scheppele 2020.

100 See, e.g., C-791/19 R *Commission v Poland* 8 April 2020, Joined Cases C-585/18, judgment of 19 November 2019. See generally L Pech and K Lane Scheppele 2017.

101 COM (2020) 1753 final, Covid-19, Guidelines for border management measures to protect health and ensure the availability of goods and essential services.

102 Coronavirus: An EU approach for efficient contact tracing apps to support gradual lifting of confinement measures, press release 16 April 2020, available at https://ec.europa.eu/commission/presscorner/detail/en/ip_20_670. Accessed 31 May 2021.

103 See e.g. N Elkin-Koren 2020.

104 https://www.reuters.com/article/us-health-coronavirus-israel-monitoring/israels-top-court-says-government-must-legislate-covid-19-phone-tracking-idUSKCN2280RN. Accessed 31 May 2021.

105 Regulation (EU) 2016/679 of the European Parliament and of the Council of 27 April 2016 on the protection of natural persons with regard to the processing of personal data and on the free movement of such data.

106 E-privacy Directive 2009/136/EC on universal service and users’ rights relating to electronic communications networks and services.

107 Case C-311/18—Schrems II, judgement of 16 July 2020.

108 C Cordelli 2019, A Dorfman & A Harel 2016.

109 A Harel 2014, K Cordelli 2020, M Thorburn 2010.

110 A Dorfman and Harel 2016.

111 M Klatt 2015.

112 Ibid.

113 B McKean 2019. On coercion and Kantian philosophy, see A Ripstein 2009.

114 I Benbaji 2020.
